# Proteome of airway surface liquid and mucus in newborn wildtype and cystic fibrosis piglets

**DOI:** 10.1186/s12931-023-02381-x

**Published:** 2023-03-16

**Authors:** Ana M. Rodriguez-Piñeiro, Florian Jaudas, Nikolai Klymiuk, Andrea Bähr, Gunnar C. Hansson, Anna Ermund

**Affiliations:** 1 Department of Medical Biochemistry and Cell Biology, Sahlgrenska Academy, University of Gothenburg, Gothenburg, Sweden; 2 Center for Innovative Animal Models, Ludwig-Maximilians-University, Munich, Germany

**Keywords:** Acute phase proteins, Airway mucus, Cystic fibrosis, Mass-spectrometry, MUC5B, MUC5AC, Protein disulfide isomerase

## Abstract

**Background:**

The respiratory tract is protected from inhaled particles and microbes by mucociliary clearance, mediated by the mucus and the cilia creating a flow to move the mucus cephalad. Submucosal glands secrete linear MUC5B mucin polymers and because they pass through the gland duct before reaching the airway surface, bundled strands of 1000–5000 parallel molecules exit the glands. In contrast, the surface goblet cells secrete both MUC5AC and MUC5B.

**Methods:**

We used mass-spectrometry based proteomic analysis of unstimulated and carbachol stimulated newborn wild-type (WT) and cystic fibrosis transmembrane conductance regulator (CFTR) null (CF) piglet airways to study proteins in the airway surface liquid and mucus, to investigate if levels of MUC5AC and MUC5B were affected by carbachol stimulation and whether the proteins clustered according to function.

**Results:**

Proteins in the first four extracted fractions clustered together and the fifth fraction contained the mucus cluster, mucins and other proteins known to associate with mucins, whereas the traditional airway surface liquid proteins clustered to fraction 1–4 and were absent from the mucus fraction. Carbachol stimulation resulted in increased MUC5AC and MUC5B.

**Conclusions:**

These results indicate a distinct separation between proteins in the washable surface liquid and the mucus fraction. In fractions 1–4 from newborn CF piglets an additional cluster containing acute phase proteins was observed, suggesting an early inflammatory response in CF piglets. Alternatively, increased levels of these proteins could indicate altered lung development in the CF piglets. This observation suggests that CF airway disease is present at birth and thus, treatment should commence directly after diagnosis.

**Supplementary Information:**

The online version contains supplementary material available at 10.1186/s12931-023-02381-x.

## Introduction

The lungs are in constant contact with the environment via inhaled air. Since the air contains various pollutants, particles, microorganisms and potential pathogens, a system must be present to clear the airways to avoid accumulation of agents causing infection or inflammation. This is achieved by mucociliary clearance, where mucus collects debris and microorganisms and the cilia of multiciliated epithelial cells create a fluid flow by coordinated beating. Specialized surface epithelial cells called goblet cells produce the mucins, in humans and pigs mainly MUC5AC but also MUC5B and mucus associated proteins in non-inflamed conditions [1, 2]. Mucus threads from surface goblet cells collect particles and clear them from the airways [3]. A major source of mucus is the submucosal gland mucous cells, which produce MUC5B. The presence of submucosal glands in human and pig conducting airways enables production of bundled mucus strands, which sweep over the airway surface to clear inhaled particles and bacteria [4–7].

The airways are covered by a thin liquid film that enables ciliary beating. Traditionally, proteomic analysis of airway surface liquid has been done by bronchoalveolar lavage [8, 9]. However, bronchoalveolar lavage fluid is a mixture of liquid secretions from alveolar and airway epithelial cells as well as mucus from surface goblet cells and submucosal glands and does not allow distinction of the different components. Understanding the different mucus and liquid components is important to understand airway protection. We have previously observed that mucus in the form of bundled strands is attached to mucus from the surface cells [1, 5]. To address which proteins are collected on the bundled strands and which are not, we compared proteins easily washed off the tracheobronchial surface with the protein composition of Alcian blue stained mucus using mass spectrometry-based proteomics. Samples collected from newborn wild-type (WT) and cystic fibrosis transmembrane conductance regulator (CFTR) null (CF) piglets revealed that distinct proteins were bound to the mucus as opposed to the ones easily washed off and associated with the airway liquid.

## Materials and methods

### Animals

Experimental procedures were performed in accordance with the EU Directive 2010/63/EU for the care and use of laboratory animals, the NIH and ARRIVE guidelines. Ethical permits for protocols were obtained from Regierungen von Oberbayern, Munich, Germany (AZ55.2-1-54-2531-78-07) and Jordbruksverket, Jönköping, Sweden (Dnr 6.7.18-12708/2019) for experiments involving newborn CFTR null (CF) and wild-type (WT) piglets (*Sus scrofa domesticus*).

### Preparation of piglet airways

Newborn piglets of both sexes were used. Breeding male and female heterozygous CF carrier animals from a previously described CF pig model [10] generated CF and WT littermate piglets. Induction of birth, genotyping, euthanasia and dissection of the airway tree were carried out as described [3]. Within 24 h of birth, airways were placed in a 50 ml tube with Perfadex® solution, pH 7.2 (XVIVO Perfusion, Gothenburg, Sweden) and shipped at 4 °C overnight to Gothenburg.

### Sample collection

**Fig. 1:**
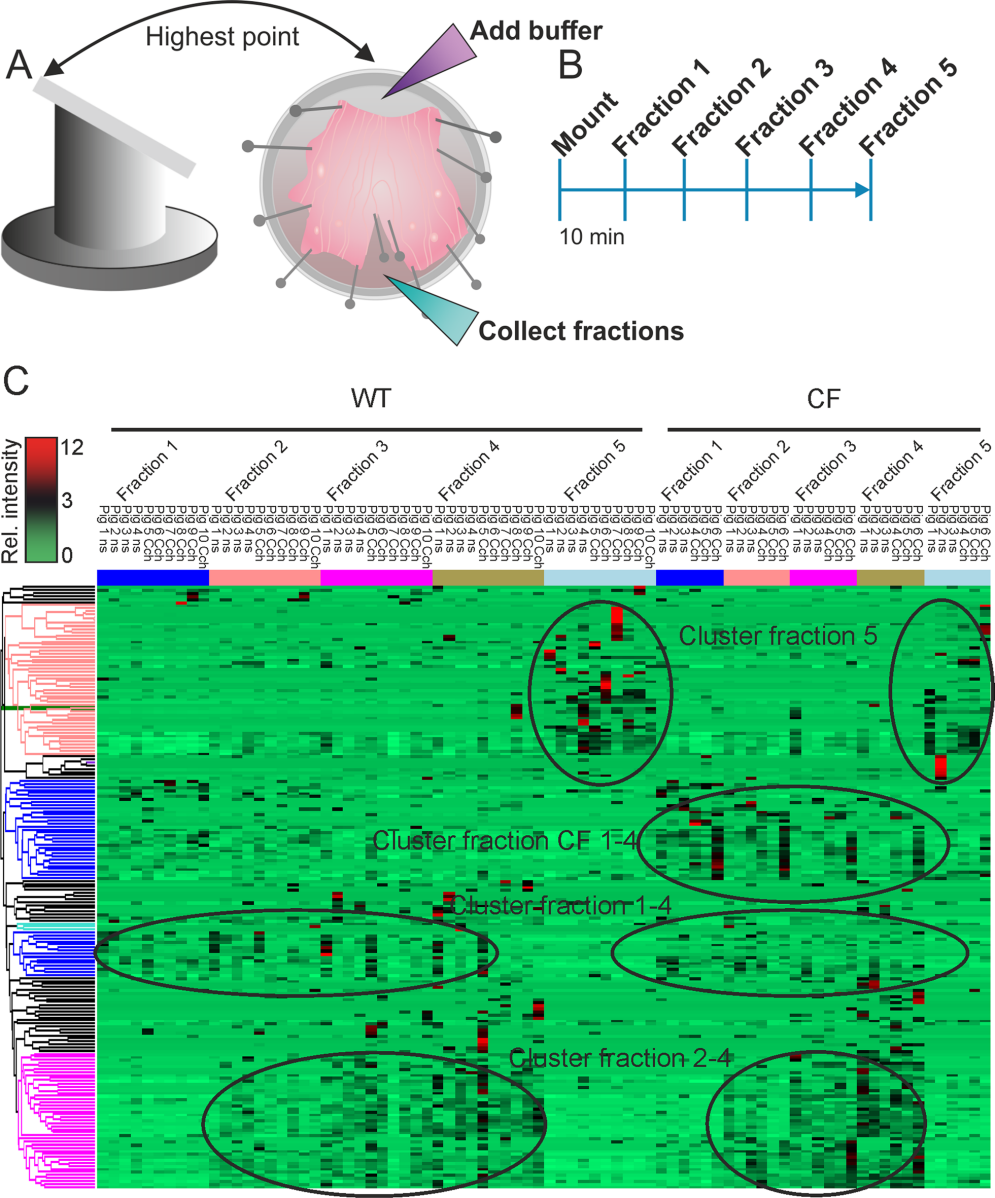
Collection of fractions for mass spectrometry based proteomic analysis. **A** Distal trachea and primary bronchi were mounted in a Petri dish and placed on a platform tilted 20º with the cephalad trachea on the highest point. Buffer was added to the proximal trachea and collected from the lowest point. **B** Five fractions were collected with 10 min interval between addition of buffer to the highest point and collection from the lowest point of the tissue. **C** Cluster analysis of mass spectrometry results generated five clusters, fraction 5, fraction 1–4, fraction 2–4 and CF piglet fraction 1–4 as indicated

The experiments were performed in explanted airway tissue from newborn CF piglets [10] and WT littermates removed and dissected directly after euthanasia of the piglets. The complete airway tree from larynx to distal bronchioles was transported overnight in Perfadex buffer. The airway tree was intact as the airway lumen was filled with air at mounting despite transport in liquid. The distal trachea and primary bronchi were excised, opened along the dorsal smooth muscle and mounted in a Sylgard-coated Petri dish, using insect needles, as described previously [5]. The mounted trachea was 30 mm in length and 10–12 mm across, with the bifurcation at the very distal end. For unstimulated samples, 500 µl aerated Krebs-Glucose buffer (116 mM NaCl, 1.3 mM CaCl_2_, 3.6 mM KCl, 1.4 mM KH_2_PO_4_, 23 mM NaHCO_3_, 1.2 mM MgSO_4_, 10 mM D-glucose, 5.1 mM Na-glutamate, and 5.7 mM Na-pyruvate, pH 7.4, gassed with 95% O_2_/5% CO_2_) was added and for stimulation with carbachol, 100 µl aerated Krebs-Glucose buffer (pH 7.4) containing 10 µM carbachol was added to the luminal surface. The tissue was incubated at 37ºC on a heating block for 10 min. Then, 400 µl aerated Krebs-Glucose buffer (pH 7.4) was added to the proximal end of the carbachol-stimulated tissue, the Petri dish placed on a platform heated to 37 °C and tilted 20º. Importantly, the cephalad end of the trachea was placed at the highest point of the tilted platform, to allow the buffer to drain from proximal trachea to bronchi (Fig. 1A). Fractions were collected from the lowest point, between the primary bronchi (Fig. 1A, B, Fraction 1). After collection, 500 µl aerated Krebs-Glucose buffer (pH 7.4) was added to the topmost part of the trachea (Fig. 1A) and fraction 2–4 were collected with 10 min intervals (Fig. 1B). After collection of fraction 4, 500 µl aerated Krebs-Glucose buffer (pH 7.4) containing 0.4 mM Alcian blue 8GX was added to the topmost part of the trachea on the tilted platform to stain the mucus, resulting in staining of bundled mucus strands and 10 min later, fraction 5 was collected by pipetting on the airway surface to aspirate the mucus.

### Mass-spectrometry analysis

The collected fractions were processed for mass spectrometry as described previously [11]. A total number of 80 samples were prepared, comprising five fractions collected out of 10 WT piglet tracheas (4 non-stimulated and 6 stimulated) and 6 CF piglet tracheas (3 non-stimulated and 3 stimulated). In short, samples were mixed overnight at 37ºC with 250 µl of reducing buffer (6 M guanidinium hydrochloride [GuHCl], 5 mM EDTA, 0.1 M DTT in 0.1 M Tris–HCl pH 8.5) and 100 µl of the reduced sample were processed by a modified filter-aided sample preparation (FASP) protocol using 6 M GuHCl [12]. The enzymes LysC (10 ng/µl, Wako Chemicals, Neuss, Germany) and trypsin (10 ng/µl, Promega, Madison, WI) were employed to digest the proteins into peptides. These were purified through C18 StageTips [13] and submitted to nano-liquid chromatography tandem mass spectrometry (nanoLC-MS/MS) on a Q-Exactive HF mass spectrometer (Thermo Fisher Scientific, Waltham, MA) under the conditions previously described [11].

The resulting raw spectra files were analyzed with MaxQuant (version 1.5.7.4, RRID:SCR_014485) against the UniProt pig proteome (version of 20180301). LysC and trypsin were set for the enzyme specificity, Cys carbamidomethylation was selected as fixed modification, and Met oxidation and protein N-terminal acetylation were ascribed to the variable modifications. The false discovery rate (FDR) was set at 1%. A total of 2328 proteins were identified and filtered, eliminating those identified only by site, reverse identifications and potential contaminants. Protein levels were calculated by normalizing individual peptide intensities to the total intensity of all identified proteins. These are presented in Additional file 2: Table S1. Proteins were filtered for presence of predicted signal sequence and lack of transmembrane domain based on information available in UniProt to identify secreted proteins and these are presented in Additional file 3: Table S2.

### Immunofluorescent staining

Tissue pieces from piglet tracheas were fixed in 4% formalin or Carnoy (60% methanol, 30% chloroform and 10% glacial acetic acid; Cat# 322415, 372978 and 695092 respectively, Sigma-Aldrich, St. Louis, MO), embedded in paraffin and cut in 4 μm thick sections. Sections were dewaxed with Xylene (Cat# 534056 Sigma-Aldrich, St. Louis, MO) and hydrated in decreasing concentrations of ethanol. Antigen retrieval was performed by heating to 100ºC for 20 min and cooling for 20 min in room temperature in 0.01 M citric buffer pH 6.0. Unspecific binding was blocked for 60 min with 3% donkey serum in Tris-buffered saline (TBS) and permeabilized with 0.1% Triton X-l00 at ambient temperature. Primary antibodies used were mouse monoclonal anti-MUC5AC clone 45M1 at 1:1000 (Cat# MA1-38224, RRID:AB_2146842, ThermoFisher Scientific, Waltham, MA), goat polyclonal anti-AGR2 (T-17) at 1:100 (Cat# sc-54561, RRID:AB_2225107, Santa Cruz Biotechnology, Dallas, TX), mouse monoclonal anti-ERp57 at 1:2000 (PDIA3, Cat# ab13506, RRID:AB_1140700, Abcam, Cambridge, UK), mouse monoclonal anti-AGP at 1:200 (ORM1, Cat# ab239473, Abcam, Cambridge, UK), were incubated sequentially overnight with custom made rabbit anti-human anti-MUC5B directed against the D3 domain at 1:60,000 or rabbit monoclonal anti-CD31 at 1:1000 (Cat# ab182981, Abcam, Cambridge, UK) in blocking solution at 4 °C. The custom-made anti-MUC5B antibody was characterized previously [14]. Secondary antibodies according to species origin of the primary antibodies were donkey anti-rabbit Alexa Fluor 488 (Cat# A32790, RRID:AB_2762833), donkey anti-goat Alexa Fluor 555 (Cat# A32816, RRID:AB_2762839, Thermo Fisher Scientific, Waltham, MA) and donkey anti-mouse Alexa Fluor 647 (Cat# A-31571, AB_162542, Thermo Fisher Scientific, Waltham, MA). Secondary antibodies were incubated in blocking solution for two hours at ambient temperature in the dark. Nuclei were counterstained with Hoechst 34580 (Cat# 565877, RRID:AB_2869723, BD Biosciences, San Jose, CA) and sections were mounted with Prolong gold mounting medium (Cat# P36934, RRID:SCR_015961, Thermo Fisher Scientific, Waltham, MA). Images were acquired with an upright LSM 900 Axio Examiner Z1 confocal imaging system, Plan-Apochromat 63x/1.4 Oil DIC M27 (Cat# 420782-9900-799), Airyscan 2 detector and Zen blue software (RRID:SCR_013672, Carl Zeiss, Oberkochen, Germany). Images were processed with Zen blue (airyscan processing) and Imaris software, version 9 (Oxford Instruments, Abingdon, U.K.).

### Statistical analysis

The Perseus software (version 1.5.6.0, RRID:SCR_015753) was employed for data filtration and clustering. Replacement of missing values was done by simulation of the normal distribution. Hierarchical clustering was performed by the Euclidean distance, with average linkage. The results were graphically plotted as a heat map. Clustered proteins are presented in Additional file 4: Table S3.

Data are presented as median with interquartile range and all data points are shown. Each piglet represents one data point, n = number of piglets, unless otherwise described in the figure legends. Pooling was performed to compare washable fractions to mucus fractions, compare WT to CF piglets or to demonstrate differences between stimulated and unstimulated fractions. Number of piglets in each group and analysis method are presented in each figure legend. Statistical analyses were performed using GraphPad Prism version 9 (RRID:SCR_002798, GraphPad, San Diego, CA). Differences were assessed with two-tailed non-parametric Mann–Whitney test for comparison of two groups or Kruskal–Wallis and Dunn’s multiple comparisons test for comparisons between multiple groups. Significance was defined as P ≤ 0.05. No methods of randomization or determination of sample size were used due to limited access to piglet tissue. The experiments were not performed blinded due to the unmistakable morphology of newborn CF piglet tracheas.

## Results

The distal trachea and proximal bronchi of less than 24 h old newborn WT and CF piglets were opened along the dorsal smooth muscle and pinned open with needles mucosal side up on a platform tilted 20º with the cephalad end at the highest point and heated to 37 ºC. Krebs-buffer (500 µl) was added to the highest (proximal) end of the trachea and allowed to drain to the lowest point of the tilted airway where fractions were collected without touching the airway surface (Fig. 1A). The liquid was collected after 10 min, the same volume of Krebs-buffer (500 µl) added to the cephalad end of the trachea and the liquid again collected after 10 min (Fig. 1B). This procedure was repeated and four airway liquid or washable fractions were collected (fraction 1–4). After collecting the fourth fraction, Alcian blue in Krebs-buffer was added and incubated for 10 min before the Alcian blue stained mucus was collected with a pipette (fraction 5). The same procedure was performed on unstimulated and carbachol (10 µM) stimulated piglet airways.

The fractions were processed for proteomics using a modified FASP protocol followed by high resolution nano-liquid chromatograph mass-spectrometry. Obtained peptides were searched against a porcine proteome data base complemented with pig mucin sequences. More than 2,000 proteins were identified and used for combined protein cluster analysis (Fig. 1C). This resulted in three distinct but similar clusters for the WT and CF piglets (1–4, 2–4 and 5) and an additional one for CF piglets. No difference in clusters was observed between unstimulated and stimulated fractions. Note that the clusters fraction 1–4, 2–4 and 5 contain the same proteins from both WT and CF piglets. The additional cluster observed in CF piglets was found in all four washable fractions, but this does not exclude that the normalized intensities could be higher in stimulated samples. All proteins identified by mass spectrometry are presented in Additional file 2: Table S1, secreted proteins are presented in Additional file 3: Table S2 and clustered proteins with a short description of their function in Additional file 4: Table S3.

Even though the lung parenchyma and smaller airways were removed before mounting, some proteins from these parts could have been secreted before the tissue was mounted and thus, some of these proteins may be detected in addition to soluble proteins from the proximal airway epithelium and submucosal glands. The Alcian blue stained mucus remained on the tracheobronchial surface during washings and was collected in the fifth fraction, reflecting mucus and proteins associated with the mucus.

### Fraction 5, the mucus fraction

**Fig. 2:**
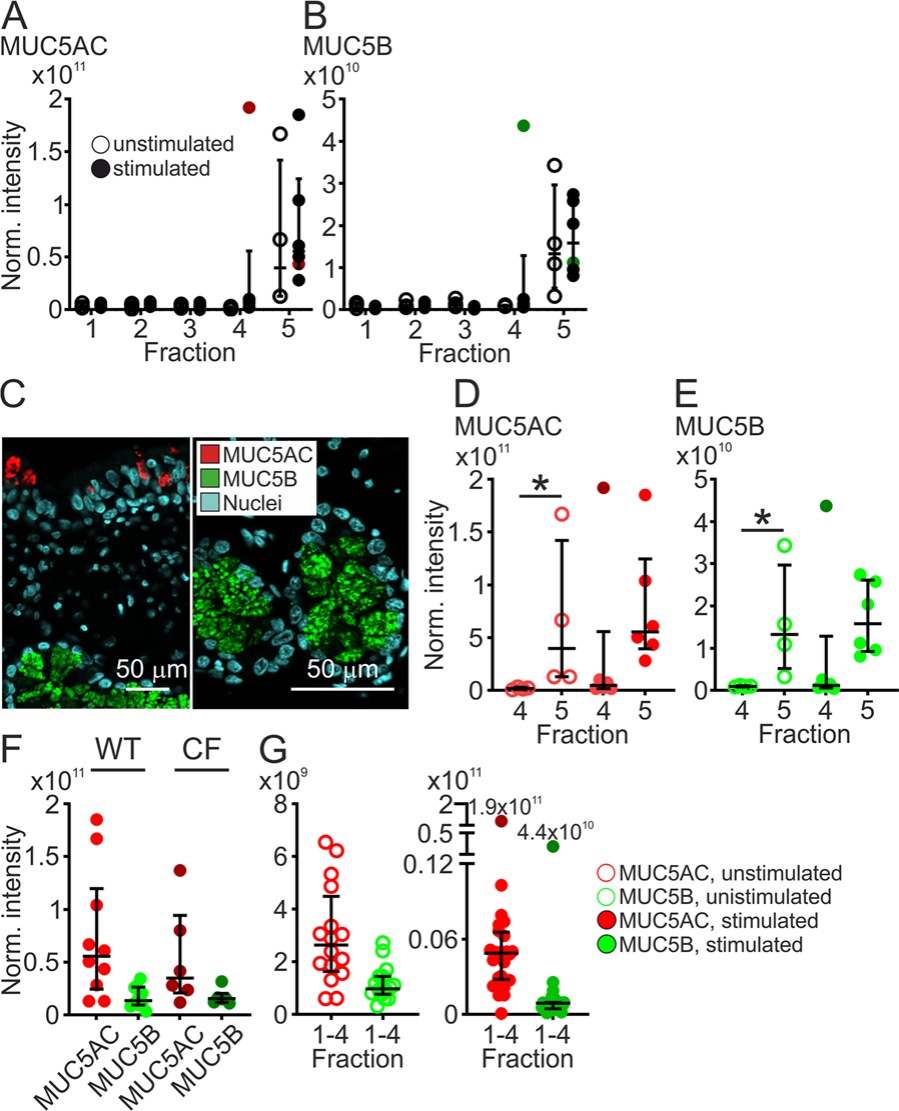
Airway gel-forming mucins were found in fraction 5, the mucus fraction. **A** Normalized intensities of MUC5AC in all fractions, unstimulated (open circles, n = 4) and stimulated (filled circles, n = 6) WT piglets. Red filled circles in fraction 4 and 5 indicate the normalized intensity in the same piglet in fraction 4 (the highest value) and fraction 5. **B** Normalized intensities of MUC5B in all fractions, unstimulated (open circles, n = 4) and stimulated (filled circles, n = 6) WT piglets. Green filled circles in fraction 4 and 5 indicate the normalized intensity in the same piglet in fraction 4 (the highest value) and fraction 5. **C** Immunostaining of paraffin sections from newborn WT piglets demonstrating that MUC5AC (red) was produced by surface epithelial goblet cells (left image) and MUC5B (green) was mainly produced by submucosal glands (right image). Scale bar left image 50 µm, scale bar right image 20 µm. **D** Comparison between normalized intensities for MUC5AC in fraction 4 and 5 for unstimulated (open circles, n = 4) and stimulated (filled circles, n = 6) WT piglets. There was more MUC5AC in unstimulated samples from fraction 5 than fraction 4, *P = 0.03, Mann–Whitney test. Dark red filled circle indicates outlier. **E** Comparison between levels of MUC5B in fraction 4 and 5 for unstimulated (open circles, n = 4) and stimulated (filled circles, n = 6) WT piglets. There was more MUC5B in unstimulated samples from fraction 5 than fraction 4, *P = 0.03, Mann–Whitney test. Dark green filled circle indicates outlier. **F** Pooling normalized intensities from unstimulated and stimulated (10 µM carbachol) WT piglets (n = 10) and CF piglets (n = 6) respectively demonstrated that there was more MUC5AC than MUC5B in fraction 5. **G** There was more MUC5AC (red open circles, n = 4, 16 values) than MUC5B (green open circles, n = 4, 16 values) in unstimulated WT piglets, pooled relative intensities from fraction 1, 2, 3 and 4. The same was found for stimulated WT piglets, pooled relative intensities from fraction 1, 2, 3 and 4 (MUC5AC red closed circles, n = 6, 24 values, MUC5B green closed circles, n = 6, 24 values). Each data point in **A**–**C**, **E**–**G** represents one piglet and results are presented with median and interquartile range. Scale bar in **C** 50 µm

As expected, the Alcian blue stained mucus collected in fraction 5 contained the two secreted gel-forming mucins MUC5AC and MUC5B. Levels of MUC5AC in both unstimulated and stimulated WT piglets were high in fraction 5 and low in fraction 1–4 (Fig. 2A). The same pattern was observed for MUC5B (Fig. 2B). Immunostainings in paraffin sections confirmed that goblet cells in the airway surface epithelium secrete MUC5AC (red) and submucosal glands secrete MUC5B (green) (Fig. 2C). Both unstimulated and stimulated piglets had higher amounts of MUC5AC in fraction 5 (mucus fraction) than fraction 4 (the last washable fraction) (Fig. 2D). Similarly, MUC5B was lower in fraction 4 than fraction 5 (Fig. 2E). However, only the difference between the unstimulated groups reached significance due to outlier values for both MUC5AC and MUC5B in the stimulated samples (dark green and red, Fig. 2A, B, F, G) originating from the same piglet, likely due to detached mucus slipping into fraction 4. Previous experiments utilizing internal standard heavy peptides for pig MUC5AC and 5B have shown that the relative intensities of the peptides for each mucin roughly reflect the relative amounts of each molecule [5]. The relative intensities of peptides for MUC5AC and MUC5B were different, indicating that the MUC5AC levels in fraction 5 was higher for both unstimulated and stimulated WT and CF piglets (Fig. 2F, G). Pooling normalized intensities in fractions 1–4 for unstimulated and stimulated WT piglets suggested more MUC5AC than MUC5B under both conditions (Fig. 2G). Together these results imply that surface goblet cell derived MUC5AC was prominent in both washable and mucus fractions and that carbachol stimulation induced secretion of predominantly MUC5AC.

**Fig. 3:**
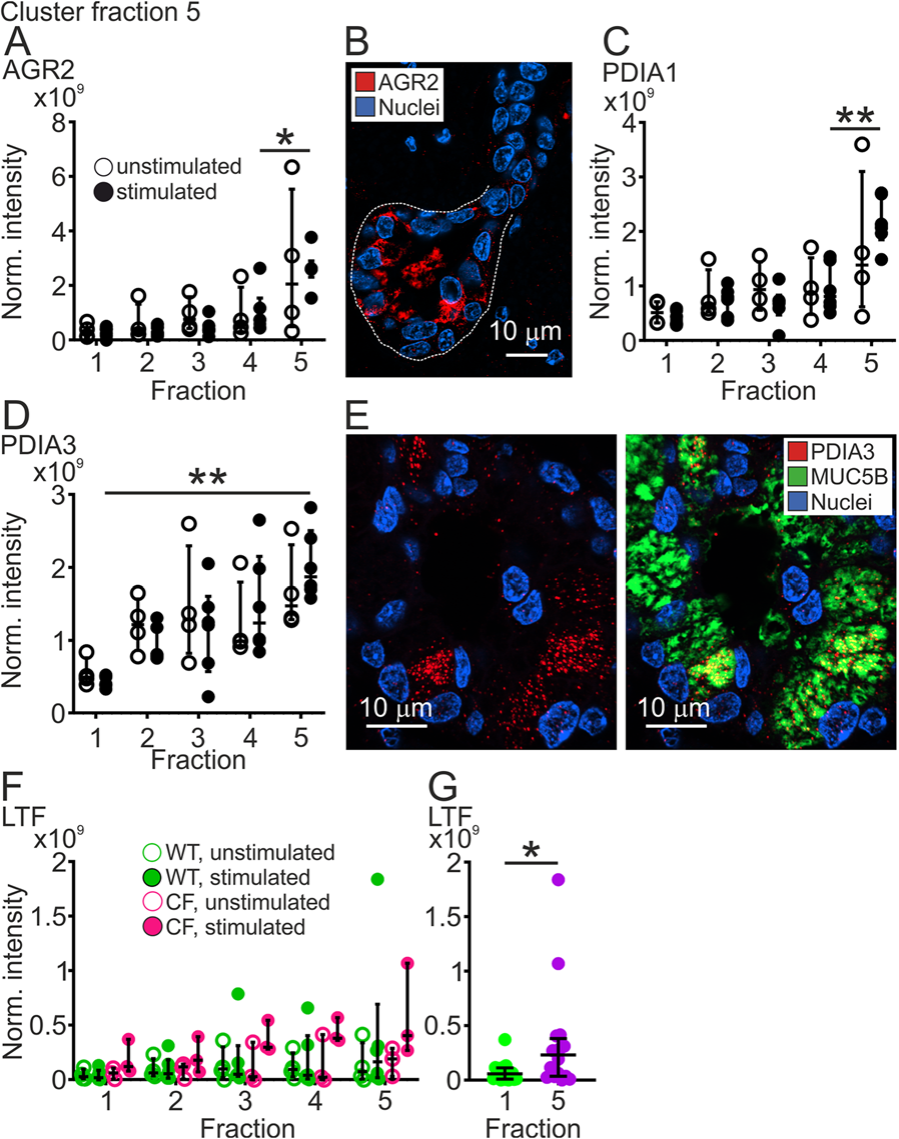
Protein disulfide isomerases clustered to fraction 5. **A** Levels of AGR2 in fraction 1–5 from unstimulated (open circles, n = 4) and stimulated (filled circles, n = 6) WT piglets. There was more AGR2 in fraction 5 than 4 in stimulated WT tracheas, *P = 0.04, Mann–Whitney test. **B** Immunostaining of paraffin sections from newborn WT piglets demonstrating that AGR2 (red) was produced in submucosal gland epithelial cells. Dashed line outlines the submucosal gland. Scale bar: 10 µm. **C** Levels of PDIA1 in fraction 1–5 from unstimulated (open circles, n = 4) and stimulated (filled circles, n = 6) WT piglets. There was more PDIA1 in fraction 5 than 4 in stimulated WT tracheas, **P = 0.004, Mann–Whitney test. **D** Levels of PDIA3 in fraction 1–5 from unstimulated (open circles, n = 4) and stimulated (filled circles, n = 6) WT piglets. There was more PDIA3 in fraction 5 from stimulated WT piglets than in fraction 1 from the same piglets, n = 6, **P = 0.002. **E** Immunostaining of paraffin sections from newborn WT piglets demonstrating that PDIA3 (red) was produced in submucosal gland MUC5B producing epithelial cells (green). Scale bar: 10 µm. **F** The antibacterial protein lactoferrin clustered to fraction 5. Normalized intensities in unstimulated WT piglets (green) denoted by open circles (n = 4 piglets) and stimulated by filled circles (n = 6 piglets) and CF piglets (pink) denoted by open circles (n = 3 piglets) and stimulated by filled circles (n = 3 piglets). **G** There was more lactoferrin in fraction 5 compared to fraction 1 when all values were pooled (WT and CF, unstimulated and stimulated, n = 16 in each group), *P = 0.03, Mann–Whitney test. Each data point in **A**, **C**, **D**, **F**, **G** represents one piglet and results are presented with median with interquartile range. Scale bar in **C** and **E** 10 µm

In addition to the two mucins, the fraction 5 cluster contained other proteins, among others the protein disulfide isomerases (PDIs), known to be important for protein assembly in the endoplasmatic reticulum. Their presence in fraction 5 suggests that they follow the mucins during secretion. One of these, anterior gradient protein 2 (AGR2; Fig. 3A), is a small protein of 175 amino acids known to be associated with intestinal [15, 16] and airway mucus [17]. In stimulated WT piglets, there was more AGR2 in fraction 5 compared to fraction 4. Tissue staining for AGR2 suggested the highest prevalence in submucosal glands (Fig. 2B, negative controls in Additional file 1: Fig. S1A and B), as has been previously demonstrated [17]. Staining in the surface epithelium was lower than in submucosal glands (Additional file 1: Fig. S2A). The protein PDIA1, the beta subunit of prolyl 4-hydroxylase, was also higher in fraction 5 than fraction 4 from stimulated WT piglets (Fig. 3C). Another PDI, PDIA3, had a slightly different pattern, with increasing levels from fraction 1 to 5 (Fig. 3D). Immunostaining demonstrated that PDIA3 was mainly present in the submucosal glands and interestingly only produced by a subset of MUC5B-positive cells (Fig. 3E, negative controls in Additional file 1: Fig. S1C–F). Surface staining was negative (Additional file 1: Fig. S2B). These PDIs were found on the airway surface, but whether they have any extracellular function is unknown. The fraction 5 cluster also contained the antimicrobial glycoprotein lactoferrin (LTF), known to be produced by submucosal glands [18]. Lactoferrin displayed similar patterns in unstimulated and stimulated WT and CF piglets (Fig. 3F). The levels of lactoferrin were higher in fraction 5 than fraction 1, when analyzing pooled unstimulated and stimulated samples from both WT and CF piglets (Fig. 3G).

### Fraction 1–4, the washable fractions

**Fig. 4:**
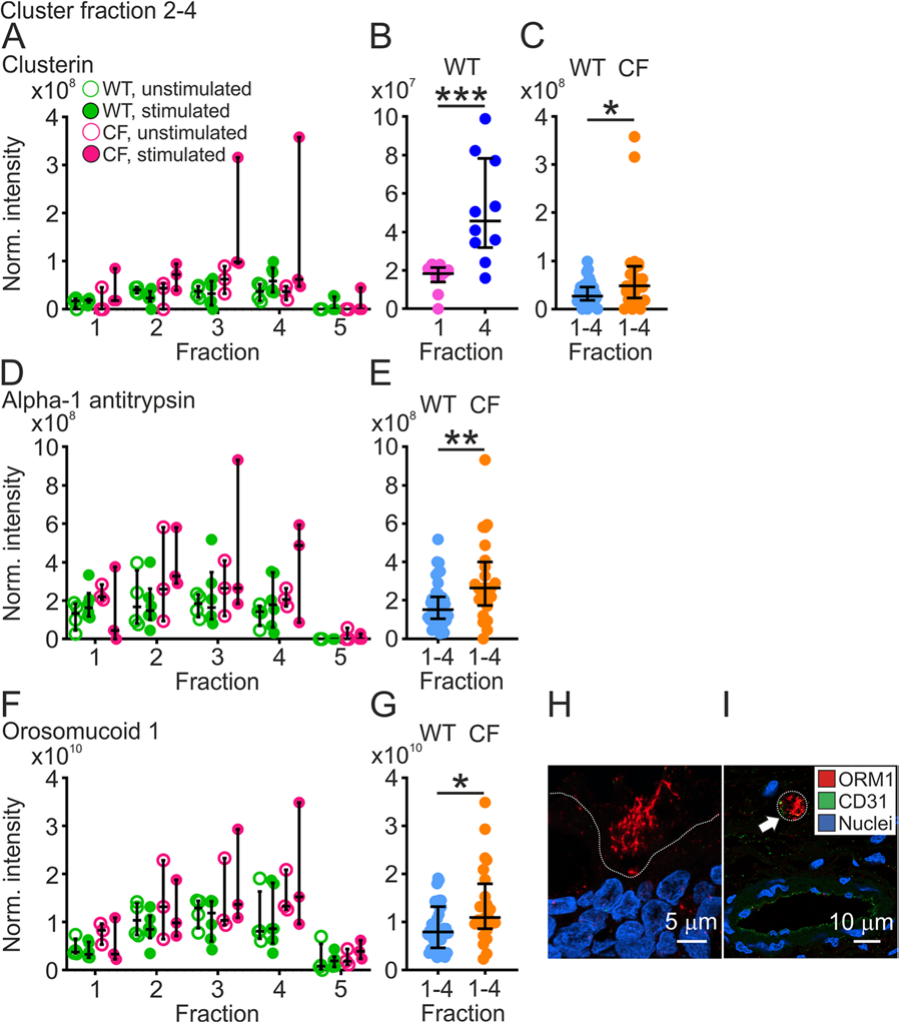
Traditional airway surface liquid proteins clustered to fraction 2–4. **A **Normalized intensities for the molecular extracellular chaperone clusterin (CLU) in unstimulated (open circles, n = 4) and stimulated (filled circles, n = 6) WT piglets (green) and unstimulated (open circles, n = 3) and stimulated (filled circles, n = 3) CF piglets (pink). **B** There was more clusterin in fraction 4 compared to fraction 1 when normalized intensities from unstimulated and stimulated WT piglets were pooled (n = 10 in each group), ***P = 0.0007, Mann–Whitney test. **C** Pooled normalized intensities from unstimulated and stimulated WT piglets, fraction 1–4 (n = 10, 40 values) and relative intensities from unstimulated and stimulated CF piglets (n = 6, 24 values) demonstrated that clusterin was higher in CF than WT piglets, *P = 0.02, Mann–Whitney test. **D** The serine protease inhibitor alpha-1 antitrypsin (SERPINA1) was found in WT piglets (green), fraction 1–4 but hardly at all in fraction 5, unstimulated samples (open circles, n = 4) and stimulated samples (filled circles, n = 6) and displayed the same pattern in CF piglets (pink) as in WT piglets, unstimulated samples (open circles, n = 3) and stimulated samples (filled circles, n = 3). **E** There was more alpha-1 antitrypsin in fraction 1–4 from CF piglets than fraction 1–4 from WT piglets, pooled values from unstimulated and stimulated piglets (WT n = 10, 40 values, CF n = 6, 24 values), **P = 0.009, Mann–Whitney test. **F** Orosomucoid 1 (ORM1) displayed a trend for increasing levels from 1–4 in WT tracheas (green), unstimulated (open circles, n = 4) and stimulated (filled circles, n = 6) and a similar trend in CF piglets (pink), unstimulated (open circles, n = 3) and stimulated (filled circles, n = 3). **G** There was more orosomucoid 1 in fraction 1–4 from CF piglets than fraction 1–4 from WT piglets, pooled relative intensities from unstimulated and stimulated piglets (WT n = 10, 40 values, CF n = 6, 24 values), *P = 0.02, Mann–Whitney test. Each data point in **A**, **B**, **D** and **F** represents one piglet and results are presented as median with interquartile range. **H** Immunostaining of paraffin sections from newborn WT piglets visualized orosomucoid 1 (red) collected on the airway surface (dashed line). Scale bar: 5 µm. **I** Immunostaining of paraffin sections from newborn WT piglets. Orosomucoid 1 (red) was detected in small capillaries (CD31, green) in the airway submucosa, indicated by white arrow and dashed line. Scale bar in **H** and **I** 10 µm

The tracheobronchial surface was sequentially washed and the collected material called washable fractions 1–4. The material was secreted from the epithelial cells or submucosal glands and these proteins were lower in the mucus fraction. The first wash differed from the rest as this likely contained proteins from more distal airways, secreted when the airway tree was still intact and transported with mucociliary transport to the distal trachea. We thus focused on the fraction 2–4 cluster (Fig. 1C), which contained the ubiquitously present extracellular molecular chaperone clusterin (CLU) with numerous suggested functions. In line with our observation, it has been found in the epithelium of human airways [19]. In both WT and CF piglets we detected an increasing trend from fraction 1 to 4, but relatively lower levels of clusterin in fraction 5 (Fig. 4A). More clusterin was found in fraction 4 compared to fraction 1 in pooled values from WT piglets (Fig. 4B), suggesting that clusterin was newly secreted by the cells in the explant or alternatively that clusterin was more tightly associated with structures on the airway surface or the mucus than proteins with higher normalized intensity in fraction 1. Slightly higher levels of clusterin were found in CF compared to WT piglets (Fig. 4C).

Alpha-1 antitrypsin (SERPINA1), an endogenous inhibitor of neutrophil elastase is produced in the lung where it controls inflammatory responses, evident from the hereditary disease alpha-1 antitrypsin deficiency [20]. The inhibitor was previously demonstrated not only in humans [21] but also in bronchoalveolar lavage fluid from newborn piglets [8]. We detected alpha-1 antitrypsin in fraction 1–4 from WT and CF piglets (Fig. 4D), but the normalized intensity was low in the mucus fraction (Fig. 4D). The pooled normalized intensity from fraction 1–4 was higher in CF than WT piglets (Fig. 4E).

The acute-phase protein orosomucoid 1 (ORM1), also called alpha-1-acid glycoprotein, clustered to fraction 2–4 and was detected in fraction 1–4 in unstimulated and stimulated WT and CF piglets. There were relatively low levels of orosomucoid 1 in the mucus fraction in both WT and CF piglets (Fig. 4F), but the normalized intensities were higher in CF than WT piglets (Fig. 4G). Immunolocalization of orosomucoid 1 identified the protein on the airway surface, but not inside the epithelial cells (Fig. 4H). The protein was also detected inside small capillaries in the airway submucosa, positive for the endothelial cell marker CD31 (Fig. 4I, negative controls in Additional file 1: Fig. S1C–F).

Further investigation of proteins clustering to fraction 1–4 revealed low levels of intracellular proteins localized to the endoplasmatic reticulum and Golgi (Additional file 2: Tables S1 and Additional file 3: S2). Examples of such proteins were calumenin, involved in CFTR maturation [22], reticulocalbin-3 from the same family, a molecular chaperone important for biosynthesis of pulmonary surfactant-associated proteins [23] and a molecular chaperone involved in collagen production called SERPINH1 or heat shock protein 47 [24]. Haptoglobin, which binds free hemoglobin to inhibit its oxidative activity [25] and porcine SERPINA3-2 or alpha-1-antichymotrypsin 2 also clustered to fraction 1–4. The closest human ortholog is alpha-1-antochymotrypsin, an acute phase protein induced during inflammation encoded by the gene SERPINA3 [26].

### CF piglets

**Fig. 5:**
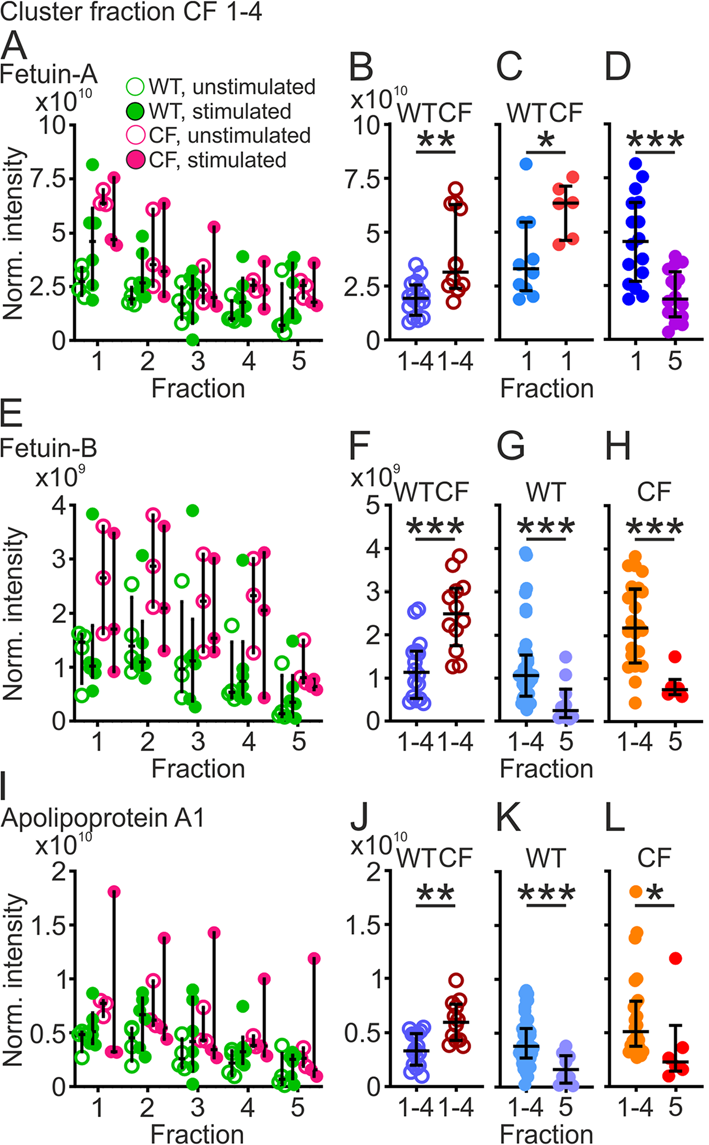
Acute phase proteins cluster to CF fractions 1–4. **A** Fetuin-A (AHSG) displayed a decreasing trend from fraction 1 to 4 in WT piglets (green), unstimulated (open circles, n = 4) and stimulated airways (filled circles, n = 6) and the downward trend was more pronounced in CF piglets (pink) unstimulated (open circles, n = 3) and stimulated (filled circles, n = 3). **B** There was more Fetuin-A in unstimulated CF (n = 3, 12 values) than WT piglets (n = 4, 16 values), pooled normalized intensities from fraction 1–4, **P = 0.002, Mann–Whitney test. **C** In fraction 1, there was more fetuin-A in CF (n = 6) than WT (n = 10) piglets, pooled normalized intensities from unstimulated and stimulated piglets, *P = 0.03, Mann–Whitney test. **D** There was more fetuin-A in fraction 1 than fraction 5, pooled normalized intensities from unstimulated and stimulated WT and CF piglets (n = 16 in each group), ***P = 0.0003, Mann–Whitney test. **E** Fetuin-B normalized intensities displayed a similar trend as fetuin-A in unstimulated (open circles, n = 4) and stimulated (filled circles, n = 6) WT piglets (green) and the same trend was observed for samples from CF piglets (pink), unstimulated (open circles, n = 3) and stimulated (filled circles, n = 3) airways. **F** Levels of fetuin-B were higher in unstimulated CF piglets (n = 3, 12 values) than WT piglets (n = 4, 16 values), ***P = 0.0004, Mann–Whitney test. **G** Fetuin-B was also higher in all airways (unstimulated and stimulated) from fraction 1–4 (n = 10, 40 values) compared to fraction 5 (n = 10) in WT piglets, ***P = 0.0005, Mann–Whitney test. **H** The same was observed for CF airway fraction 1–4 (n = 6, 24 values) compared to CF airway fraction 5 (n = 6), ***P = 0.0005, Mann–Whitney test. **I** The pattern of apolipoprotein A1 normalized intensity in the fractions most closely resembled fetuin-A in WT piglets (green), unstimulated (open circles, n = 4) and stimulated airways (filled circles, n = 6) and the pattern was similar in CF piglets (pink) with a downward trend from fraction 1 to 5, unstimulated (open circles, n = 3) and stimulated tracheas (filled circles, n = 3). **J** Levels of apolipoprotein A1 were higher in unstimulated CF piglets (n = 12) than WT piglets (n = 16), **P = 0.002, Mann–Whitney test. **K** Apolipoprotein A1 was also higher in all airways (unstimulated and stimulated) from fraction 1–4 (n = 10, 40 values) compared to fraction 5 (n = 10) in WT piglets, ***P = 0.0008, Mann–Whitney test. **L** The same was observed for CF fraction 1–4 (n = 6, 24 values) compared to fraction 5 (n = 6), *P = 0.02, Mann–Whitney test. Each data point in **A**, **C**, **D**, **E** and **I** represents one piglet and results are presented as median with interquartile range

The washable fractions from CF piglets contained proteins forming an additional cluster not present in fraction 1–4 (Fig. 1C). This cluster included fetuin-A (alpha-2-Heremans Schmid-glycoprotein, AHSG), fetuin-B (FETUB) and apolipoprotein A1 (APOA1). Fetuin-A decreased from fraction 1 to 4 (Fig. 5A). The normalized intensities of pooled fraction 1–4 were higher in unstimulated CF than WT piglets (Fig. 5B), also observed in pooled fraction 1 (Fig. 5C). Furthermore, the levels were lower in fraction 5 compared to fraction 1 (Fig. 5D). The normalized intensity for fetuin-B followed a similar pattern to fetuin-A (Fig. 5E) with higher levels in CF than WT piglets (Fig. 5F) and also more fetuin-B in fractions 1–4 than fraction 5 of both WT and CF piglets (Fig. 5G, H). Apolipoprotein A1 also had a similar distribution pattern as fetuin-A (Fig. 5I) and with relatively more apolipoprotein A1 in CF than WT piglets (Fig. 5J) and more in fractions 1–4 than fraction 5 (Fig. 5K, L).

## Discussion

Using tracheobronchial explants from newborn piglets, we successfully identified proteins present in the non-attached washable fractions of airway liquid, which were well separated from the attached mucus. This upper part of the pig airways has abundant submucosal glands and is anatomically and functionally similar to human proximal airways. Several of the identified proteins have previously been identified in human bronchioalveolar lavage fluid and airway surface liquid [8, 27], suggesting that the proteins identified here were secreted from the tissue and not detected due to poor tissue integrity. Neither the peripheral airways nor the lung parenchyma was included, largely excluding proteins from these locations and thus most proteins typical for bronchioalveolar lavage fluid. For example, one of the most abundant proteins in human and mouse bronchoalveolar lavage fluid is uteroglobin (SCGB1A1 or CCSP), normally found in distal bronchi but not in proximal bronchi or submucosal glands [11, 28, 29]. Uteroglobin was not found in any of the fractions as it is not produced in the trachea [28, 29], demonstrating that most of the proteins identified here are produced in the distal trachea.

The protein signatures in the washable fractions were different from the mucus fraction, indicating that a majority of the mucus and associated proteins are attached to the airway surface and not detached by the gentle procedures used here. The mucus was recovered as Alcian blue positive material by pipetting and we know from previous experiments that this material largely consists of the bundled strands, previously called bundles [1, 5, 30]. The core of the bundled strands was the MUC5B mucin made in the submucosal glands. After secretion they become coated with material from the surface cells, including the MUC5AC mucin [5]. In fraction 5 containing the Alcian blue stained material we identified MUC5AC and MUC5B and there was more MUC5AC than MUC5B in the WT piglets. The origin of MUC5AC is the surface goblet cells and not the submucosal glands, implying that the surface goblet cells contribute more significantly to the bundled mucus strands than expected. Stimulation of WT piglet airways with carbachol resulted in increased MUC5AC compared to unstimulated WT airways in fraction 4, further supporting that the washable fractions contain surface liquid proteins, whereas fraction 5 was mainly derived from the submucosal glands. However, comparing proteins identified in our samples to the proteome of submucosal gland secretions in humans [31], we identified MUC5B and lactoferrin (LTF) in the mucus fraction, but alpha-1 antitrypsin and orosomucoid 1 clustered to the soluble fractions. We detected alpha-1 antitrypsin and orosomucoid 1 in the mucus fraction, but their levels were higher in the washable fractions than in the mucus fraction.

In addition to the secreted gel-forming mucins in fraction 5, several proteins of the PDI and chaperone families were observed. The PDIs are normally localized to the endoplasmatic reticulum lumen, where they help to form correct disulfide bonds and function as molecular chaperones [32]. Association between PDIs and gel-forming mucins is not surprising as the mucins contain numerous cysteines and during folding they have to find the correct disulfide bonding partner. More surprising was that they were secreted as most should be retained via their C-terminal retention signal (KDEL or a variant) [33]. However, a number of molecular chaperones and PDIs have been found extracellularly in the mucus [34]. One of these is AGR2, a small protein with a single cysteine found together with MUC2 in intestinal mucus [16]. AGR2 was also observed in airway surface cells producing MUC5AC and MUC5B and mucous cells in airway submucosal glands producing MUC5B. Allergen challenge of mice lacking AGR2 caused endoplasmatic reticulum stress and the unfolded protein response, but its detailed function still remains incompletely understood [17]. We detected AGR2 in piglet airways together with other PDIs clustering to the mucus fraction. Immunofluorescent labeling indicated AGR2 mainly in the submucosal glands, emphasizing that fraction 5 proteins were secreted mainly from the submucosal glands. We observed the same profile also for PDIA1 and PDIA3 with higher levels in fraction 5. The antibacterial protein lactoferrin was linked to human disease as levels were increased in people with cystic fibrosis and chronic obstructive pulmonary disease (COPD) [11, 35] and also clustered to fraction 5, consistent with its production in submucosal glands [18]. In contrast, we detected comparatively low levels of traditional airway surface liquid proteins such as fetuin-A, fetuin-B, orosomucoid 1, alpha-1 antitrypsin and apolipoprotein A1 in fraction 5, indicating that proteins do not randomly gather on the mucins [8].

The washable fraction clusters contained proteins generally believed to be airway surface liquid proteins such as clusterin, alpha-1 antitrypsin and orosomucoid 1. Many cell types produce clusterin, also known as apolipoprotein J (APOJ), serum protein 40, 40, sulfated glycoprotein 2 and complement lysis factor [36]. Clusterin clustered to fraction 2–4 and was higher in fraction 4 than 1, suggesting that it was secreted from the surface cells of the explant. Clusterin is an extracellular chaperone as it prevents aggregation of proteins by binding to exposed hydrophobic regions of partially unfolded proteins and promotes endocytosis and lysosomal degradation [37–39]. Clusterin has been observed in goblet cells in normal lungs and the levels were increased in bronchioalveolar lavage fluid from never smokers compared to smokers [11, 19]. Alpha-1 antitrypsin encoded by the SERPINA1 gene is the most abundant serine protease inhibitor (serpin) in human plasma and deficiency is known to cause lung disease [40]. Although alpha-1 antitrypsin clustered to the same fraction as clusterin, the pattern was different as there was no difference in levels of alpha-1 antitrypsin between fraction 1 and 4, suggesting less new secretion compared to clusterin or constitutive secretion of alpha-1 antitrypsin and progressive release of clusterin after sequential washes.

Orosomucoid 1 or alpha-1-acid glycoprotein belongs to the same group of positive acute phase proteins as alpha-1 antitrypsin and is distributed to various body fluids such as mucus [41]. In newborn piglets, 50% of total serum protein was orosomucoid 1, whereas the corresponding number was 0.3% in adult pigs, indicating that the level of orosomucoid 1 decreases dramatically after birth [42]. The protein was also induced in alveolar type II cells during both acute and chronic pulmonary inflammation [43]. Fetuin-A and fetuin-B clustered to CF piglet fraction 1–4 but are considered to be negative acute phase proteins as fetuin-A decreased in patients suffering from systemic inflammation due to bacterial infections, as opposed to alpha-1 antitrypsin and orosomucoid 1, which were upregulated in these patients [44] and fetuin-B mRNA was decreased in inflamed rats [45]. The fact that the fetuins are increased instead of decreased may be an indication that development is altered in CF piglets.

Although respiratory tract manifestations of cystic fibrosis are the leading cause of morbidity and mortality, it is not fully understood if the respiratory tract is normal at birth or when lung disease begins. Several attempts to answer these questions have been made, but for obvious ethical and technical reasons, studies cannot be conducted on newborn humans. Nevertheless, results from infants below six months of age indicate that lung disease begins as early as within the first weeks after birth with structural changes, inflammatory responses and infection [46]. Mucus accumulation and airway mucus plugging are part of cystic fibrosis pathophysiology and the total mucus concentration as well as the staining intensity for MUC5AC and MUC5B were elevated in bronchioalveolar lavage fluid from children with cystic fibrosis compared to age matched controls. Furthermore, mucin concentration correlated with inflammatory markers (neutrophil counts and interleukin 8 concentration) but not with bacterial burden, indicating that mucus accumulation was present also in the absence of bacterial infection [47].

Further study of cystic fibrosis lung disease and the origin of inflammation is not possible in humans and thus, CFTR knock-out (CF) pigs and ferrets have been engineered. Newborn CF piglets examined 6–12 h after birth displayed no histopathological abnormalities and there were no differences in leukocyte cell counts or interleukin 8 concentration in bronchiolar lavage fluid from CF compared to non-CF piglets [10, 48, 49]. There was also no evidence of infection, but the number of cultured bacteria in lung homogenates from CF piglets was higher than in non-CF piglets, indicating defective mucus transport and bacterial killing already at birth [49, 50]. Additional evidence that inflammation in cystic fibrosis is directly linked to CFTR dysfunction was contributed by the observation that pharmacological inhibition of CFTR in air–liquid interface cultures of human tracheal epithelial cells for three days resulted in increased interleukin 8 production, indicating that impaired CFTR function is enough to induce an inflammatory response [51]. To further investigate this, one future direction is to link protein levels to function and we are in the process of doing pathway analysis of both airway epithelial mRNA and secreted proteins.

In the present study, we could compare WT and CF piglets. Proteins in CF piglets clustered as in WT piglets, except that we found an additional protein cluster in CF piglets. There was no difference in mucin normalized intensities between WT and CF piglets, in line with the observation that CF piglets do not overproduce mucus [10, 49]. Instead, levels of clusterin, alpha-1 antitrypsin, orosomucoid 1 and apolipoprotein A1 were increased in CF piglet samples from fraction 1–4 compared to the corresponding WT piglet samples. Furthermore, fetuin-A clustered together with apolipoprotein A1 to fraction 1–4 from CF piglets and both were higher in CF than WT piglets. Even though increased levels of clusterin, alpha-1 antitrypsin and orosomucoid 1 in CF piglets could indicate that their development is altered compared to WT piglets, the fact that apolipoprotein A1 was also increased indicates that because acute phase proteins produced in the liver were increased in newborn CF piglets, they have mounted a systemic inflammatory response, further strengthening the hypothesis that inflammation is a result of stagnant mucus and lack of CFTR function, not bacterial infection [52].

## Conclusions

The collection method enables detection of relevant proteins present in human bronchoalveolar lavage fluid, but with the added benefit that mucus proteins could be separated from the washable fractions. Furthermore, even though newborn CF piglets do not show overt signs of inflammation, our results suggest that inflammation promoting mechanisms are activated already at birth.

## Supplementary Information

**Additional file 1: Figure S1.** Immunostaining controls. (A) Submucosal gland. Secondary antibody donkey anti-goat Alexa Fluor 555, no primary antibody. Scale bar: 20 µm. (B) Airway surface epithelium. Secondary antibody donkey anti-goat Alexa Fluor 555, no primary antibody. Scale bar: 20 µm. (C) Submucosal gland. Secondary antibody donkey anti-mouse Alexa Fluor 555, no primary antibody. Scale bar: 20 µm. (D) Airway surface epithelium. Secondary antibody donkey anti-mouse Alexa Fluor 555, no primary antibody. Scale bar: 20 µm. (E) Submucosal gland. Secondary antibody donkey anti-rabbit Alexa Fluor 488, no primary antibody. Scale bar: 10 µm. (F) Airway surface epithelium. Secondary antibody donkey anti-rabbit Alexa Fluor 488, no primary antibody. Scale bar: 10 µm. Nuclei counterstained with Hoechst in A-F. **Figure S2.** Epithelial staining relating to figure 3. (A) Staining with AGR2 (red) and nuclei (blue) in the surface epithelium of the same tissue used in figure 3B. Scale bar: 10 µm. (B) Staining with PDIA3 (red), the MUC5B mucin (green) and nuclei (blue) in the surface epithelium of the same tissue used in figure 3E. Scale bar: 10 µm. Dashed lines delineate the epithelium (ep). Cilia (ci) and goblet cell (gc) are indicated.

**Additional file 2: Table S1.** Relative intensities for all proteins.

**Additional file 3: Table S2.** Relative intensities for secreted proteins.

**Additional file 4: Table S3.** Proteins identified in the different clusters and description of their function.

## Data Availability

The datasets generated and analyzed in the current study are available from the authors upon reasonable request.

## Abbreviations

AGR2: Anterior gradient protein 2
CFTR: Cystic fibrosis transmembrane conductance regulator
CF: CFTR null piglet
COPD: Chronic obstructive pulmonary disease
FASP: Filter-aided sample preparation
GuHCl: Guanidinium hydrochloride
nanoLC-MS/MS: Nano-liquid chromatography tandem mass spectrometry
PDI: Protein disulfide isomerase
SCGB1A1: Uteroglobin
TBS: Tris-buffered saline
WT: Wild-type
