# Supplementary material for: Proteome of airway surface liquid and mucus in newborn wildtype and cystic fibrosis piglets
Source: Respir Res. 2023 Mar 16;24:83. doi: 10.1186/s12931-023-02381-x (PMC10022022; doi:10.1186/s12931-023-02381-x)

Additional File 1

**Proteome of airway surface liquid and mucus in newborn wildtype and cystic fibrosis piglets**

Ana M. Rodriguez-Piñeiro^1^, Florian Jaudas^2^, Nikolai Klymiuk^2^, Andrea Bähr^2^, Gunnar C. Hansson^1^, and Anna Ermund^1^

**Author Affiliations:**

^1^Department of Medical Biochemistry and Cell Biology, Sahlgrenska Academy, University of Gothenburg, Gothenburg, Sweden

^2^Center for Innovative Animal Models, Ludwig-Maximilians-University, Munich, Germany.

**Corresponding Author:** Anna Ermund, telephone: +46-31-7860000, fax: +46-31- 7862150, e-mail: [Anna.Ermund@medkem.gu.se](mailto:Anna.Ermund@medkem.gu.se), ORCID ID: 0000-0002-3233-043X

**Additional Figures**

**Figure S1. Immunostaining controls.** *(A)* Submucosal gland. Secondary antibody donkey anti-goat Alexa Fluor 555, no primary antibody. Scale bar: 20 µm. *(B)* Airway surface epithelium. Secondary antibody donkey anti-goat Alexa Fluor 555, no primary antibody. Scale bar: 20 µm. *(C)* Submucosal gland. Secondary antibody donkey anti-mouse Alexa Fluor 555, no primary antibody. Scale bar: 20 µm. *(D)* Airway surface epithelium. Secondary antibody donkey anti-mouse Alexa Fluor 555, no primary antibody. Scale bar: 20 µm. *(E)* Submucosal gland. Secondary antibody donkey anti-rabbit Alexa Fluor 488, no primary antibody. Scale bar: 10 µm. *(F)* Airway surface epithelium. Secondary antibody donkey anti-rabbit Alexa Fluor 488, no primary antibody. Scale bar: 10 µm. Nuclei counterstained with Hoechst in A-F.


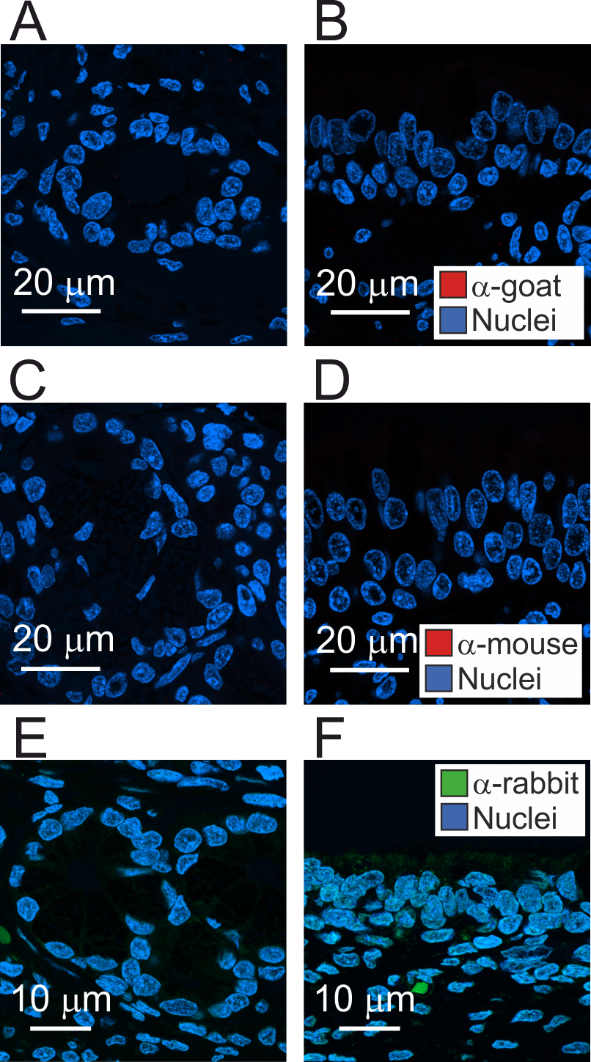


**Figure S2. Epithelial staining relating to figure 3.** *(A)* Staining with AGR2 (red) and nuclei (blue) in the surface epithelium of the same tissue used in figure 3B. Scale bar: 10 µm. *(B)* Staining with PDIA3 (red), the MUC5B mucin (green) and nuclei (blue) in the surface epithelium of the same tissue used in figure 3E. Scale bar: 10 µm. Dashed lines delineate the epithelium (ep). Cilia (ci) and goblet cell (gc) are indicated.


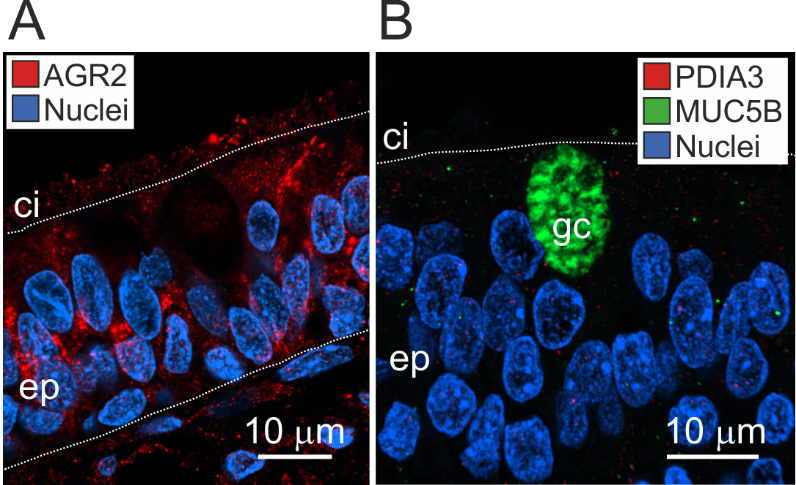

Supplement: Supplementary file 1 — Additional file 1: Figure S1. Immunostaining controls. (A) Submucosal gland. Secondary antibody donkey anti-goat Alexa Fluor 555, no primary antibody. Scale bar: 20 µm. (B) Airway surface epithelium. Secondary antibody donkey anti-goat Alexa Fluor 555, no primary antibody. Scale bar: 20 µm. (C) Submucosal gland. Secondary antibody donkey anti-mouse Alexa Fluor 555, no primary antibody. Scale bar: 20 µm. (D) Airway surface epithelium. Secondary antibody donkey anti-mouse Alexa Fluor 555, no primary antibody. Scale bar: 20 µm. (E) Submucosal gland. Secondary antibody donkey anti-rabbit Alexa Fluor 488, no primary antibody. Scale bar: 10 µm. (F) Airway surface epithelium. Secondary antibody donkey anti-rabbit Alexa Fluor 488, no primary antibody. Scale bar: 10 µm. Nuclei counterstained with Hoechst in A-F. Figure S2. Epithelial staining relating to figure 3. (A) Staining with AGR2 (red) and nuclei (blue) in the surface epithelium of the same tissue used in figure 3B. Scale bar: 10 µm. (B) Staining with PDIA3 (red), the MUC5B mucin (green) and nuclei (blue) in the surface epithelium of the same tissue used in figure 3E. Scale bar: 10 µm. Dashed lines delineate the epithelium (ep). Cilia (ci) and goblet cell (gc) are indicated. [file 12931_2023_2381_MOESM1_ESM.docx]
