# Supplementary material for: Proteome of airway surface liquid and mucus in newborn wildtype and cystic fibrosis piglets
Source: Respir Res. 2023 Mar 16;24:83. doi: 10.1186/s12931-023-02381-x (PMC10022022; doi:10.1186/s12931-023-02381-x)
Supplement: Supplementary file 4 — Additional file 4: Table S3. Proteins identified in the different clusters and description of their function. [file 12931_2023_2381_MOESM4_ESM.docx]

**Table S3. Proteins identified in the different clusters and description of their function.**

|  | Gene name | Protein name | Function |
| --- | --- | --- | --- |
| Fraction 5 | APOC3 | Apolipoprotein C-III | Inhibits lipoprotein lipase and hepatic lipase |
|  | NPG1 | Protegrin-1 | Broad-spectrum antimicrobial peptide |
|  | RETN | Resistin | Peptide hormone secreted by immune and epithelial cells |
|  | AZU1 | Azurocidin | Azurophilic granules or lysosomes of neutrophils, contains at least 10 different substances for microorganism killing |
|  | LOC100154047 | Unknown |  |
|  | SCGB3A1 | Secretoglobin family 3A member 1 | Cytokine-like |
|  | **LTF** | Lactotransferrin/lactoferrin | Antimicrobial |
|  | LOC100512873 | Unknown |  |
|  | PR39 | Antibacterial protein PR39 | Antimicrobial (porcine) |
|  | MPO | Myeloperoxidase | Stored in azurophilic granules, peroxidase, antimicrobial |
|  | PMAP23 | Antibacterial peptide PMAP23 | Antimicrobial activity against both Gram-positive and negative bacteria |
|  | NPG3 | Protegrin-3 | Antimicrobial, leukocytes and epithelial cells. Human LL-37 |
|  | CDH13 | Cadherin-13/T-cadherin | Cell adhesion |
|  | UGGT1 | UDP-glucose:glycoprotein glucosyltransferase 1 | Recognize misfolded glycoproteins in the ER |
|  | ALCAM | Cluster of differentiation 166 | Cell survival |
|  | NENF | Neudesin/Neuron-derived neurotrophic factor | Neurotrophic factor |
|  | A0A287B5M9 | Unknown |  |
|  | **AGR2** | Anterior gradient 2 | PDI |
|  | P4HA1 | Prolyl 4-hydroxylase subunit alpha-1 | Collagen synthesis |
|  | PPT1 | Palmitoyl-protein thioesterase 1 | Lysosomal degradation |
|  | MAN2B1 | Lysosomal alpha-mannosidase | Catabolism of N-linked carbohydrates |
|  | CTSS | Cathepsin S | Lysosomal cysteine protease, degrading antigen for presenting by MHC class II. Elastase function in alveolar macrophages. |
|  | SUMF2 | Sulfatase modifying factor 2 | ER lumen |
|  | ERLIN2 | ER lipid raft associated 2 | Mediating ER-associated degradation of activated IP3 receptors |
|  | CNPY3 | Protein canopy homolog 3 | Toll-like receptor-specific co-chaperone for HSP90B1 |
|  | RPN1 | Dolichyl-diphosphooligosaccharide--protein glycosyltransferase subunit 1 | Protein N-glycosylation |
|  | SSR2 | Translocon-associated protein subunit beta | Regulation of ER retention by calcium binding |
|  | MIA3 | Transport and Golgi organization protein 1 homolog | Protein export from the ER (cargo too large for COPII-vesicles) |
|  | TXNDC12 | Thioredoxin domain-containing protein 12 | Thiol-disulfide oxidase activity (PDI), defense against oxidative stress |
|  | CANX | Calnexin | ER membrane molecular chaperone |
|  | MANF | Mesencephalic astrocyte-derived neurotrophic factor | Luminal endoplasmic reticulum |
|  | ERP29 | Endoplasmic reticulum resident protein 29 | Processing of secretory proteins in the endoplasmic reticulum |
|  | PPIB | Peptidylprolyl isomerase B | Cyclophilin, regulates protein folding and maturation |
|  | BPIFB1 | BPI fold-containing family B member 1/LPLUNC1 | Immunity |
|  | **MUC5AC** | Mucin-5AC |  |
|  | **MUC5B** | Mucin-5B |  |
|  | CLCA1 | Calcium-activated chloride channel regulator 1 |  |
|  | CLCA1 |  |  |
|  | SPON1 | Spondin-1 | Cell adhesion |
|  | ERP44 | Endoplasmic reticulum resident protein 44 | Retention in the early secretory pathway, inhibits calcium channel activity of IP3R1 |
|  | SCP2 | Sterol carrier protein 2 | Intracellular transport of sterol and other lipids |
|  | CHID1 | Chitinase domain containing 1 | Carbohydrate metabolism? |
|  | CNPY2 | Canopy FGF signaling regulator 2 | Regulation of LDL-receptor activity |
|  | PRKCSH | Glucosidase 2 subunit beta | Regulatory subunit of glucosidase II, N-glycan metabolism |
|  | **PDIA3** | Protein disulfide-isomerase A3 | PDI, interacts with calreticulin and calnexin |
|  | **P4HB** | Prolyl 4-hydroxylase subunit beta | PDI, oxidoreductase |
|  | PDIA4 | Protein disulfide-isomerase A4 | PDI |
|  | GANAB | Glucosidase II alpha subunit | Protein folding and quality control, ER-resident |
|  | pdi-p5 | Protein disulfide-isomerase A6 | PDI, chaperone, inhibits aggregation of misfolded proteins |
|  | WFDC2 | WAP four-disulfide core domain protein 2 | Protease inhibitor, pulmonary epithelial cells |

| Cluster | Gene name | Protein name | Function |
| --- | --- | --- | --- |
| Fraction CF 1, 2, 3, 4 | SPP1 | Secreted phosphoprotein 1/osteopontin | Chemotactic, cell adhesion, wound healing, immune modulator |
|  | F1SG31 | Lipocln_cytosolic_FA-bd_dom domain-containing protein | Odorant binding |
|  | DCN | Decorin | Proteoglycan, related to biglycan, component of connective tissue, interacts with fibronectin |
|  | CD44 | Cluster of differentiation 44 | Cell adhesion, migration, interacts with hyaluronic acid, osteopontin, collagens, MMPs, effector-memory T-cells |
|  | PCSK1N | Proprotein convertase subtilisin/kexin type 1 inhibitor | Regulated secretory pathway |
|  | CYTL1 | Cytokine-like protein 1/C17 | Expressed in bone marrow and cord blood mononuclear cells with CD34 |
|  | MGP | Matrix Gla protein | Binds calcium, vitamin K2 dependent |
|  | SFTPD | Surfactant protein D | Collectin, innate immune system, interacts with DMBT1 |
|  | AGER | Advanced glycosylation end product-specific receptor | Mediator of acute and chronic vascular inflammation, interacts with S100A12 on endothelium, mononuclear phagocytes and lymphocytes, generation of proinflammatory mediators |
|  | BGN | Biglycan | Mineralization of bone, interacts with collagen |
|  | LOC100513767 | Unknown |  |
|  | CD5L | CD5 antigen-like | Regulator of lipid synthesis, early response to microbial infection, participates in metabolic disease |
|  | CHRDL2 | Chordin like 2 | Regulates cartilage formation |
|  | WAP | Whey acidic protein | Limited to milk - contamination |
|  | A0A287APT2 | Unknown |  |
|  | SERPINF2 | Alpha-2-antiplasmin | Serine protease inhibitor, inhibits plasmin, neutrophil elastase |
|  | VTN | Vitronectin | Hemopexin family, binds to serpins |
|  | **FETUB** | Fetuin-B | Cystatin superfamily of cysteine protease inhibitors, response to systemic inflammation |
|  | **AHSG** | alpha-2-HS-glycoprotein, Fetuin-A | Downregulates adiponectin production by adipocytes, carrier protein, inhibits precipitation of calcium and phosphate |
|  | **APOA1** | Apolipoprotein A1 | Major component of HDL particles, PGI2-stabilizing factor |
|  | LOC396685 | Unknown |  |
|  | LOX | Lysyl oxidase | Catalyzes the conversion of lysine molecules into aldehydes, form cross-links in extracellular matrix proteins |
|  | APOE | Apolipoprotein E | Cholesterol metabolism, inhibits the classical complement pathway |
|  | PROC | Autoprothrombin IIA/Protein C | Vitamin K-dependent serine protease, blood coagulation factor XIX |
|  | F12 | Coagulation factor XII, Hageman factor | Serine protease, coagulation cascade |
|  | F2 | Prothrombin | Coagulation factor II, thrombin serine protease, converts fibrinogen (soluble) into fibrin (insoluble) |
|  | P04119 | Unknown |  |
|  | MSTN | Myostatin | Inhibits muscle cell growth |
|  | HGFAC | Hepatocyte growth factor activator | Serine protease, homologous to plasminogen |
|  | KNG1 | Kininogen-1 | Precursor to high-molecular-weight kininogen, low-molecular-weight kininogen and bradykinin |
|  | CFI | Complement factor I | Regulates complement activation |
|  | APOH | Apolipoprotein H | Anticoagulation, not in lipoproteins |

| Cluster | Gene name | Protein name | Function |
| --- | --- | --- | --- |
| Fraction 1, 2, 3, 4 | FMOD | Fibromodulin | Sequence homology with biglycan and decorin, collagen fiber assembly |
|  | CILP2 | Cartilage intermediate layer protein 2 | Cartilage extracellular matrix |
|  | C1S | Complement component 1s | Classical pathway, esterase |
|  | AFM | Afamin | Serum transport |
|  | LOC106504545 | Unknown |  |
|  | ACAN | Aggrecan | Chondroitin sulfate proteoglycan family, cartilage extracellular matrix |
|  | CALU | Calumenin | Calcium-binding, localized in the ER, protein folding and sorting |
|  | HP | Haptoglobin | Binds free hemoglobin, inhibits its oxidative activity |
|  | LOC396684 | Unknown |  |
|  | LOC396685 | Unknown |  |
|  | HYOU1 | Hypoxia up-regulated protein 1 | HSP70 family, molecular chaperone, protein folding in the ER, secretion |
|  | SERPINA3-2 | Serpin A3-2 | Serine protease inhibitor |
|  | APOA4 | Apolipoprotein A4 | Chylomicron particles |
|  | RCN3 | Reticulocalbin-3 | Molecular chaperone, calcium binding, required for biosynthesis and transport of pulmonary surfactant-associated protein A, pulmonary surfactant-associated protein D and the lipid transporter ABCA3 |
|  | IGFBP6 | Insulin-like growth factor-binding protein 6 | Various |
|  | HPX | Hemopexin/beta-1B-glycoprotein | Detoxification of free heme |
|  | A0A287B5G0 | Unknown |  |
|  | SERPINH1 | Serpin H1/HSP47 | Chaperone for collagen |

| Cluster | Gene name | Protein name | Function |
| --- | --- | --- | --- |
| Fraction 2, 3, 4 | IGFBP7 | Insulin-like growth factor-binding protein 7 | Various |
|  | BPIFB2 | BPI fold-containing family B member 2/LPLUNC2 | Lipid transfer/lipopolysaccharide binding protein family |
|  | RCN2 | Reticulocalbin 2 | Calcium-binding protein in the lumen of the ER |
|  | CPB2 | Carboxypeptidase B2 | Reduces fibrinolysis |
|  | HAPLN1 | Hyaluronan and proteoglycan link protein 1 | Stabilizes interactions between extracellular matrix molecules versican and hyaluronan, facilitates fibroblast proliferation and conversion to myofibroblasts |
|  | SERPINA5 | Serpin A5 | Coagulation, heparin-dependent serine protease inhibitor |
|  | COL12A1 | Collagen alpha-1(XII) chain | Cartilage extracellular matrix |
|  | ISLR | Immunoglobulin superfamily containing leucine-rich repeat protein | Mesenchymal stem cell marker, stabilizes canonical Wnt signaling, promotes skeletal muscle regeneration |
|  | CALR | Calreticulin/ERp60 | Calcium-binding chaperone, promotes folding, oligomeric assembly and quality control in the endoplasmic reticulum |
|  | HSP90B1 | Heat shock protein 90 beta family member 1 | ATP-metabolizing molecular chaperone, localized to melanosomes and the endoplasmic reticulum |
|  | FGG | Fibrinogen gamma chain | Together with fibrinogen alpha (FGA) and fibrinogen beta (FGB), polymerizes to form an insoluble fibrin matrix |
|  | FGB | Fibrinogen beta chain | Together with fibrinogen alpha (FGA) and fibrinogen beta (FGB), polymerizes to form an insoluble fibrin matrix |
|  | COL2A1 | Collagen alpha-1(II) chain | Component of type II collagen |
|  | COL3A1 | Collagen alpha-1(III) chain | Extracellular matrix, alpha 1 chain of type III collagen |
|  | HRG | Histidine-rich glycoprotein | Immunity, coagulation |
|  | CST3 | Cystatin C/Cystatin 3 | Biomarker of kidney function |
|  | C7 | Complement component 7 | Innate immunity, part of the MAC, makes hole in pathogen surface |
|  | P3H1 | Prolyl 3-hydroxylase 1 | Basement membrane-associated chondroitin sulfate proteoglycan, prolyl 3-hydroxylase (collagen IV and V) |
|  | RPLP2 | Ribosomal protein lateral stalk subunit P2/60S acidic ribosomal protein P2 | Elongation step of protein synthesis |
|  | GPC1 | Glypican-1 | Cell surface heparan sulfate proteoglycan |
|  | CFD | Complement factor D | Chymotrypsin family of serine peptidases, adipokine, immunity, alternative complement pathway |
|  | SERPINF1 | Serpin F1 | Neurotrophic protein, no serine protease inhibitory activity |
|  | PCOLCE | Procollagen C-endopeptidase enhancer 1 | Drives the enzymatic cleavage of type I procollagen |
|  | COL6A3 | Collagen alpha-3(VI) chain | serine-type endopeptidase inhibitor activity |
|  | SERPINC1 | Antithrombin-III | serine protease inhibitor, regulates coagulation cascade |
|  | COL11A1 | Collagen alpha-1(XI) chain | controls lateral growth of collagen II fibrils |
|  | EFEMP1 | EGF-containing fibulin-like extracellular matrix protein 1 | Binds the EGF receptor |
|  | OGN | Osteoglycin/Mimecan | Keratan sulfate proteoglycan/ectopic bone formation |
|  | B4GALNT1 | Beta-1,4 N-acetylgalactosaminyltransferase 1 | Ganglioside formation |
|  | CD14 | Cluster of differentiation 14 | Innate immunity |
|  | C4A | Complement C4-A | Classical complement pathway/histamine release from mast cells, basophils |
|  | MYOC | Myocilin | Various |
|  | ICA | Inhibitor of carbonic anhydrase | Inhibits carbonic anhydrase 2 |
|  | **SERPINA1** | Alpha-1-antitrypsin | Inhibitor of serine proteases, primary target elastase, but also binds plasmin and thrombin |
|  | **CLU** | Clusterin | Prevents stress-induced aggregation of blood plasma proteins, extracellular chaperone |
|  | C3 | Complement component 3 | Innate immunity, alternative complement pathway |
|  | AFP | Alpha-fetoprotein | Fetal analog of serum albumin |
|  | **ORM1** | Alpha-1-acid glycoprotein 1 | Acute phase plasma protein |
|  | CFB | Complement factor B | Innate immunity, alternative complement pathway |
|  | ITIH1 | Inter-alpha-trypsin inhibitor heavy chain H1 | Carrier of hyaluronan in serum/hyaluronan binding to other matrix proteins, calcium binding, serine-type endopeptidase inhibitor |
|  | FCN2 | Ficolin-2 | Carbohydrate binding, opsonic activity |
|  | CFH | Complement factor H | Modulates complement activation, alternative complement pathway |
|  | PRDX4 | Peroxiredoxin-4 | Thiol-specific peroxidase, cell protection against oxidative stress |
|  | ITIH4 | Inter-alpha-trypsin inhibitor heavy chain H4 | Type II acute-phase protein, inflammatory responses to trauma |
|  | A0A075B7I5 | Unknown |  |
